# Exploring biological basis of Syndrome differentiation in coronary heart disease patients with two distinct Syndromes by integrated multi-omics and network pharmacology strategy

**DOI:** 10.1186/s13020-021-00521-3

**Published:** 2021-10-26

**Authors:** Gaosong Wu, Jing Zhao, Jing Zhao, Nixue Song, Ningning Zheng, Yuanyuan Zeng, Tingting Yao, Jingfang Zhang, Jieqiong Weng, Mengfei Yuan, Hu Zhou, Xiaoxu Shen, Houkai Li, Weidong Zhang

**Affiliations:** 1 Institute of Interdisciplinary Integrative Medicine Research, Shanghai University of Traditional Chinese Medicine, No. 1200 Cai Lun Road, Pudong New District, Shanghai, 201203 China; 2 Dongzhimen Hospital Affiliated to Beijing University of Chinese Medicine, No. 5 Haiyuncang, Dongcheng District, Beijing, 100700 China; 3 Department of Analytical Chemistry and CAS Key Laboratory of Receptor Research, Shanghai Institute of Materia Medica, Chinese Academy of Sciences, Shanghai, 201203 China; 4 University of Chinese Academy of Sciences, Beijing, 100049 China; 5 Department of Phytochemistry, School of Pharmacy, Second Military Medical University, No. 325 Guo He Road, Yangpu District, Shanghai, 200433 China

**Keywords:** Coronary heart disease, Syndrome differentiation, Traditional Chinese Medicine, Proteomics, Metabolomics, Network pharmacology

## Abstract

**Background:**

Traditional Chinese Medicine (TCM) is distinguished by Syndrome differentiation, which prescribes various formulae for different Syndromes of same disease. This study aims to investigate the underlying mechanism.

**Methods:**

Using a strategy which integrated proteomics, metabolomics study for clinic samples and network pharmacology for six classic TCM formulae, we systemically explored the biological basis of TCM Syndrome differentiation for two typical Syndromes of CHD: Cold Congealing and Qi Stagnation (CCQS), and Qi Stagnation and Blood Stasis (QSBS).

**Results:**

Our study revealed that CHD patients with CCQS Syndrome were characterized with alteration in pantothenate and CoA biosynthesis, while more extensively altered pathways including D-glutamine and D-glutamate metabolism; alanine, aspartate and glutamate metabolism, and glyoxylate and dicarboxylate metabolism, were present in QSBS patients. Furthermore, our results suggested that the down-expressed PON1 and ADIPOQ might be potential biomarkers for CCQS Syndrome, while the down-expressed APOE and APOA1 for QSBS Syndrome in CHD patients. In addition, network pharmacology and integrated analysis indicated possible comorbidity differences between the two Syndromes, that is, CCQS or QSBS Syndrome was strongly linked to diabetes or ischemic stroke, respectively, which is consistent with the complication disparity between the enrolled patients with two different Syndromes. These results confirmed our assumption that the molecules and biological processes regulated by the Syndrome-specific formulae could be associated with dysfunctional objects caused by the Syndrome of the disease.

**Conclusion:**

This study provided evidence-based strategy for exploring the biological basis of Syndrome differentiation in TCM, which sheds light on the translation of TCM theory in the practice of precision medicine.

**Supplementary Information:**

The online version contains supplementary material available at 10.1186/s13020-021-00521-3.

## Highlights

1. Our work was based on clinical samples rather than pure data analysis or animal models.

2. We conducted multiple omics studies. Especially, as for metabolomics study, we performed both untargeted and targeted metabolomics experiments.

3. We performed network pharmacological study to cross-validated the results of multi-omics study. Although the data sources of network pharmacology were completely unrelated with our omics data, they came to the same conclusion about the difference of the two Syndromes.

4. In the network pharmacological study, we made efforts to collect and screen high-quality data. We collected data from multiple TCM databases and conducted drug likeness screening. Especially, we added quality markers of each herb, whose pharmacological relevance had been validated. To enhance the reliability of targets, for each Syndrome, we only studied common targets of 3 different TCM formulae prescribed for this Syndrome.

**Supplementary Information:**

The online version contains supplementary material available at 10.1186/s13020-021-00521-3.

## Background

Traditional Chinese medicine (TCM) has developed over thousands of years in China, in which the medication is mainly practiced in the form of TCM formula based on Syndrome (ZHENG in Chinese) differentiation by TCM physicians [1, 2]. Syndrome differentiation (Bian Zheng Lun ZHI in Chinese) means comprehensive analysis of clinical information from standpoint of TCM, including pulse manifestation, tongue appearances, clinical indicators, and symptoms. Usually, same disease diagnosed by orthodox medical methods could have different TCM Syndromes. For example, in TCM diagnosis, rheumatoid arthritis is classified as the types of cold Syndrome and heat Syndrome [3, 4], coronary heart disease is categorized as several Syndromes, including heart-blood stasis, Qi stagnation and blood stasis, Qi deficiency and blood stasis, as well as Cold Congealing and Qi Stagnation [5]. Hence different TCM formulae were prescribed for different Syndromes of the same disease. Correct TCM Syndrome differentiation is the most important basis for prescribing TCM formulae. A pioneer study has illustrated the features of hot and cold Syndrome of arthritis in the context of neuro-endocrine-immune network [4]. However, it is still an enormous challenge for elucidating the scientific basis of TCM Syndrome differentiation in the context of modern biomedical science.

Coronary heart disease (CHD) lists the first position in mortality in the world, which includes four classes, i.e. nonobstructive coronary atherosclerosis, unstable angina pectoris, stable angina pectoris, and acute myocardial infarction [6]. Meanwhile, several types of typical TCM Syndromes have been well-established in TCM theory, such as Cold Congealing and Qi Stagnation (CCQS), and Qi Stagnation and Blood Stasis (QSBS), etc. [5, 7]. Although the patient stratification of CHD with different Syndromes is practical for “precise therapy” with diversified formula in TCM, the scientific basis for Syndrome differentiation of CHD is poorly understood so far. Considering that a TCM formula is prescribed according to a specific Syndrome, we think that the molecules and biological processes regulated by the formula could be associated with dysfunctional molecules and biological processes caused by the Syndrome of the disease. Hence, investigating the mechanism of the TCM formula may facilitate the illustration for the scientific basis of TCM Syndrome differentiation. Network pharmacology has been widely and effectively applied in the study for the mode of action of TCM formulae [8–10]. In this sense, the strategy of systems biology is highly valued for its potential in elucidating the mechanism of TCM Syndrome by integrating multi-omics approaches and network pharmacology [5, 11–13]. For example, Ding et al. investigated the holistic mechanism of Ge-Gen-Qin-Lian decoction in LPS-induced acute lung injury mice by using a systems biology strategy including transcriptomics, metabolomics and network pharmacology, in which a novel PI3K/Akt signaling pathway was predicted and validated [12]. Previously, we systematically explored the mechanisms underlying the 8 clinically used TCM formula for the treatment of CHD patients with different Syndromes by using network pharmacology and machine learning [5]. In the study, a series of common and Syndrome-related signaling pathways and molecular targets of CHD were determined. However, the computation-based analytic results were not experienced experimental validation.

In our current study, an integrated multi-omics and network pharmacology strategy was proposed for elucidating the scientific basis of patient stratification of TCM Syndrome in a group of patients with CHD who were also diagnosed as either CCQS or QSBS Syndrome by TCM physicians. Between August 2018 and December 2019, we collected serum samples of 111 participants, including 44 CHD patients with CCQS Syndrome, 37 with QSBS Syndrome and 30 healthy people from the Cardiology Clinic of Dongzhimen Hospital affiliated to Beijing University of Chinese Medicine and the neighboring communities in Beijing. The blood samples were then used in metabolomics and proteomics studies. Pathways involved in the pathologic process of the two Syndromes were discovered by functional annotation of differential proteins and metabolites. For each of the two Syndromes, we selected 3 classic TCM formulae clinically used for the treatment of the Syndrome. The common putative targets of the 3 formulae were considered as feature targets of the Syndrome and used for network pharmacological study and integrated analysis with omics data. At last, we identified key biological processes and molecules that may associated with the occurrence and development of the two Syndromes of CHD.

## Materials and methods

### Integrated multi-omics and network pharmacology research strategy

**Fig. 1:**
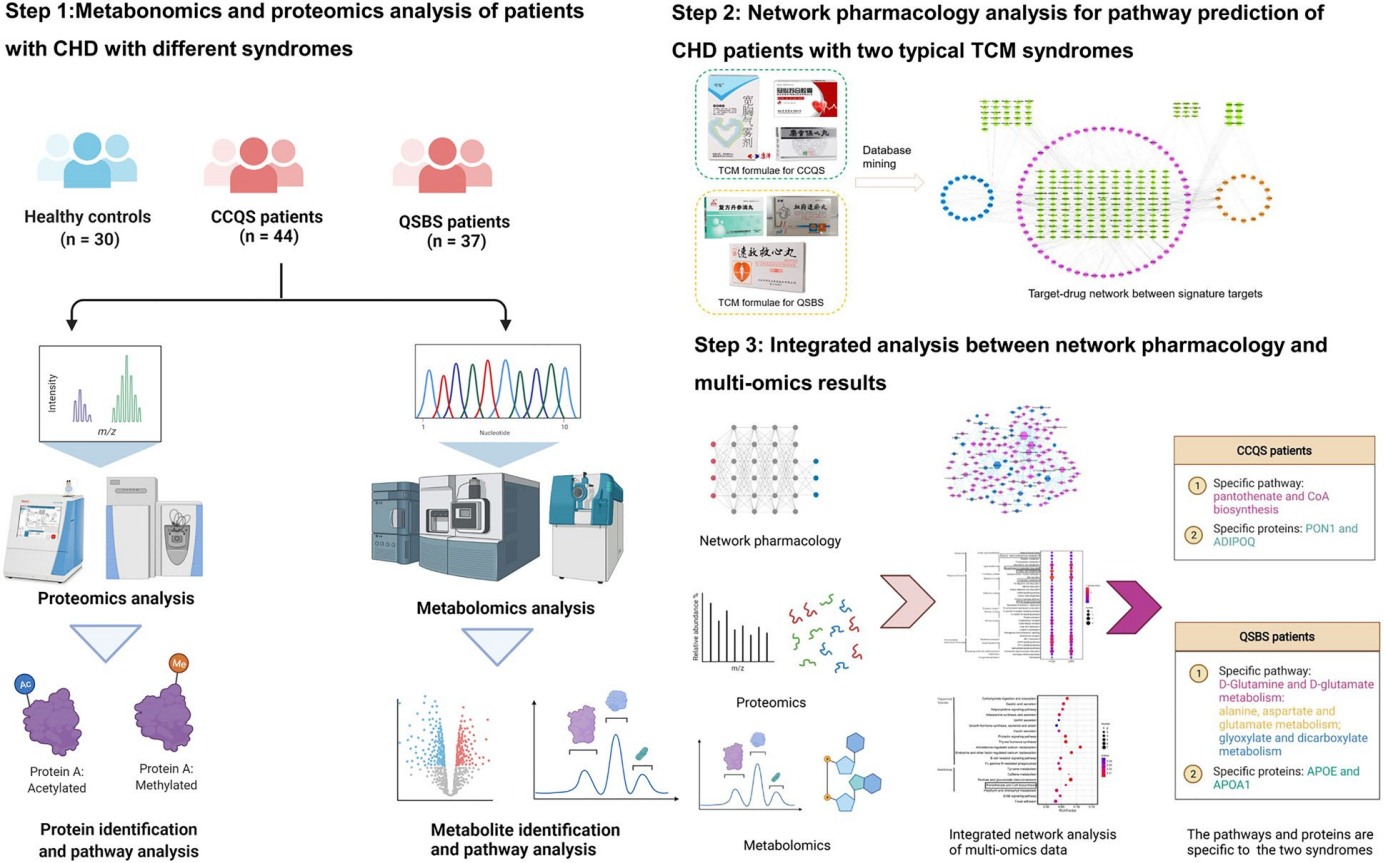
The flowchart of the integrated multi-omics and network pharmacology strategy of our current study

The workflow of the study is illustrated in Fig. 1. First, clinical CHD patients were enrolled who were diagnosed as either CCQS or QSBS Syndrome by TCM physicians simultaneously. Serum samples of patients with either CCQS or QSBS and healthy controls were analyzed with metabolomics and proteomics. Second, network pharmacology was performed based on 6 typical TCM formulae which were extensively used for the treatment of either CCQS or QSBS Syndrome of CHD patients in clinic. Third, a cross validation was performed between network pharmacology and results from both metabolomics and proteomics to characterize the basis of both CCQS and QSBS Syndromes of CHD patients.

### Participants

This study was conducted in compliance with the Declaration of Helsinki and the requirements of clinical trials by the Drug Administration Law of the People's Republic of China in Dongzhimen Hospital, Beijing between August 2018 and December 2019. The protocol and informed consent were reviewed and approved by the Beijing University of Chinese Medicine Clinical Trials and Biomedical Ethics Committee (No. DZMEC-KY-2019–121). Informed consent was obtained from all participants and their privacy rights were always preserved. All patients were enrolled by the Cardiology Clinic of Beijing University of Chinese Medicine or the surrounding communities in Beijing. Whether it meets the inclusion criteria (CCQS and QSBS Syndrome of CHD) is determined by an expert (Xiaoxu Shen, Chief Physician of Cardiovascular Medicine Dept in Dongzhimen Hospital), with verification by two assistants (Yuanyuan Zeng and Jing Zhao).

### Diagnosis criteria

Diagnostic criteria for CHD referred to the “Diagnostic Criteria for Coronary Heart Disease” defined by the International Society of Cardiology and Association and the World Health Organization in 1979: (1) With a history of typical angina pectoris or myocardial infarction, except for valvular heart disease, coronary embolism and cardiomyopathy; (2) Patients over the age of 40 for men and over 45 for women, the ECG myocardial ischemia in resting state, or the treadmill exercise test is positive, without other reasons (various heart disease, autonomic dysfunction, significant anemia, obstructive lung Emphysema, taking digitalis, electrolyte disturbances); (3) Among the risk factors of coronary heart disease, two of the three items of hypertension, diabetes and hyperlipidemia can be clinically diagnosed for coronary heart disease. Or coronary angiography or coronary CT scan shows that at least one main branch vessel has a stenosis greater than 50% to confirm the diagnosis of coronary heart disease. In this study we mainly selected patients with stable angina while satisfying the diagnosis of coronary heart disease. They are characterized by paroxysmal squeezing pain or suffocation in the anterior chest, mainly located at the back of the sternum, which can radiate to the anterior heart area and left upper limb. The ulnar side usually occurs when the labor load increases and lasts for a few minutes. The pain disappears after resting or using nitrate ester preparations. The degree, frequency, nature, and predisposing factors of pain episodes did not change significantly within weeks to months.

#### Diagnosis of TCM syndrome

According to the “Guiding Principles for Clinical Research of New Chinese Medicines”, the diagnostic criteria for Syndromes were determined as follows:

Diagnostic criteria for CCQS Syndrome: primary symptoms include chest tightness, chest pain. First set of secondary symptoms includes the condition or pain aggravates on rainy days or cold, face pale, like warmth and fear of cold, cold limbs, cramps and pain. Second set of secondary symptoms includes heart palpitations, fullness of the chest and flanks, fullness of the abdominal abdomen. The tongue is pale or dark, and the tongue coating is white. Late or tight pulse.

Diagnostic criteria for QSBS Syndrome: primary symptoms include chest tightness, chest pain. First set of secondary symptoms includes that the condition is related to emotions, emotional depression, irritability, sigh, and chest fullness; The second set of secondary symptoms includes palpitations, rough, dry, and hyperkeratotic skin, sublingual varicose veins, dark purple face and lips, dark purple tongue or petechiae, and astringent pulse.

Those who have one of the primary symptoms, one or more of the first and second set of secondary symptoms at the same time, and the tongue and pulse conditions are consistent, can be diagnosed. In the diagnosis process, three researchers (at least one senior professional title) independently differentiated syndromes at the same time and then determined the types of syndromes together.

#### Included criteria

Patients meeting the diagnostic criteria were potentially eligible for the study if they meet the following criteria: (1) age at 18 to 85 years old; (2) clearly diagnosed as angina pectoris, and are classified as CCQS or QSBS in the chest pain of TCM after the doctor's differentiation witness; (3) the included patients have stable vital signs, clear consciousness, and certain expression skills; (4) voluntary submission of written informed consent prior to enrollment.

#### Excluded criteria

Patients would be excluded if they meet one of the following criteria: (1) those who have had myocardial infarction within 3 months or have undergone coronary revascularization; (2) patients with other serious heart diseases: such as valvular heart disease, hypertrophic cardiomyopathy, severe arrhythmia (such as rapid atrial fibrillation, II–III degree atrioventricular block, etc.), severe cardiac insufficiency; (3) patients with severe liver and kidney dysfunction; (4) pregnant and lactating women; (5) those who use traditional Chinese medicine decoctions and related preparations within 2 weeks; (6) those who have participated in other drug clinical trials within 2 weeks.

### Proteomics study

#### Sample preparation

Serum proteins were prepared by in solution digestion method. All samples were randomly divided into 11 experimental groups. A standard sample was added to each experimental group and prepared at the same time. We processed one set of samples (including a standard sample) per day and all samples and standard samples were prepared within 11 days. In brief, lysis solution was added into the collected human serum samples. Then DTT and IAA were added. After this procedure, we added precipitation buffer and placed it in -20 ℃ refrigerator overnight. The cold concentrate containing proteins was centrifugated and then removed the supernatant. Pre-cooled 100% acetone was added into the precipitated proteins and removed by centrifugating. Then precooling 70% ethanol was added into the proteins and the procedure was same as before. The above two steps were repeated twice. Proteins were resuspended with 100 mM NH_4_HCO_3_ solution and digested with trypsin (Promega, USA). Peptides were collected with 10 kDa filter (Millipore Corporation, USA).

#### Protein digestion

Equal volume of all human serum samples was pooled for the generation of a spectral library. The pooled sample was depleted by High-Select™ Top14 Abundant Protein Depletion Resin (Thermo Fisher). The sample with abundant proteins removed was further digested by trypsin with the in-solution digestion method. Digested peptides were desalted and fractionated through the high-pH reverse phase liquid chromatography using a Waters XBridge BEH300 C18 3.5 μm 2.1 × 150 mm column on Agilent 1200 LC instrument using an 85-min gradient.

#### Online nanoflow LC–MS/MS analysis

EASYnLC 1000 HPLC system (Thermo Fisher Scientific) and Q Exactive HF mass spectrometer (Thermo Fisher Scientific) were used for LC–MS/MS analysis. The collected peptides were separated on a home-made column (75 × 200 mm, packed with 3.0 μm ReproSil-Pur C18 beads, Dr. Maisch GmbH, Ammerbuch, Germany). Each fractionation of peptides for spectral library generation was analyzed with data dependent acquisition (DDA) mode and separated with a 120-min gradient. For DIA analysis, desalted peptides concentration was measured by NanoDrop 2000 (Thermo Scientific). Then peptides were separated on a 70-min LC gradient. The DIA acquisition scheme consisted of 32 variable windows ranging from 350 to 1600 m/z.

#### Data analysis

DDA data for spectral library generation were analyzed via MaxQuant software (http://maxquant.org/, version 1.6.7.0). The results were imported into Spectronaut 14.0 (Biognosys) for library generation. And the generated library contained 1,095 proteins and 11,871 precursors. For human serum, DIA data was processed with Spectronaut 14.0. Statistical analysis was performed in R software. Significantly differential proteins were determined using Student’s t-test with a p-value < 0.05 and fold change (FC) > 1.1.

### Untargeted Metabolomics study

#### Sample preparation

An aliquot of 50 µl of thawed serum sample was deproteinized with 150 µl of MeOH: ACN (1:1, *v/v*) precooled to − 20 ℃. After vortex mixed for 30 s and sonication for 10 min in an ice bath, samples were overnight at − 20 ℃ to improve protein precipitation and then centrifuged at 12 000×*g* for 15 min at 4 ℃, 2 µl of supernatant was subjected to HPLC-QTOF-MS/MS analysis.

#### HPLC-QTOF-MS/MS analysis

Sample analysis was performed on a Shimadzu Nexera XR LC-20AD HPLC system equipment with SCIEX Triple TOF 5600^+^. In order to capture serum metabolic characteristics as comprehensively as possible, two different types of chromatographic columns [ACQUITY UPLC BEH amide column (2.1 × 150 mm, 1.7 µm) and ACQUITY UPLC BEH C_18_ column (2.1 × 100 mm, 1.8 µm)] were used for untargeted metabolomics analysis.

#### Data processing and analysis

The raw data were imported to the Progenesis QI for peak alignment to obtain the peak area list and the identification result list. The nonparametric univariate method (Mann–Whitney-Wilcoxon test) was used to analysis metabolites that differed in abundance between the different subgroups corrected for false discovery rate (FDR) to ensure that the peak of each metabolite was reproducibly detected in the samples. Metabolites selected as biomarker candidates for further statistical analysis were identified on the basis of variable importance in the projection (VIP) threshold of 1 from the tenfold cross-validated OPLS-DA model, which was validated at a univariate level with FDR < 0.05. The online HDMB data (https://hmdb.ca/), LIPIDMAPS (https://www.lipidmaps.org/), KEGG (https://www.kegg.jp/) and METLIN (https://metlin.scripps.edu) were used to align the molecular mass data to identify metabolites.

### Targeted metabolomics study

#### Sample preparation

An aliquot of 25 µl of thawed serum sample was added to the pre-chilled 96-well plate, then added 100 µl of methanol containing internal standard (IS) vortex for 5 min, and finally centrifuged at 4000×*g* for 30 min at 4 ℃. The supernatant (30 µl) was transferred into a new 96-well plate containing 20 µl of derivatization reagent, and the mixture was reacted at 30 ℃ for 60 min. After reaction, we added 350 µl 50% methanol solution precooled to -20 ℃ for 20 min, then centrifuged at 4000 × g for 30 min at 4 ℃, the supernatant (135 µl) was transferred into a new 96-well plate containing 15 µl of IS. Gradient dilutions of the derivatized standard stock solution were added to the left hole, and finally the plate was sealed for LC–MS analysis.

#### UPLC-ESI–MS/MS analysis

Chromatographic analysis was performed using a Waters ACQUITY I-Class UPLC equipped with an ACQUTIY UPLC BEH C18 column (2.1 × 100 mm, 1.7 µm). Waters Xevo TQ-S triple quadrupole mass spectrometer was combined with UPLC system via the electro-spray ionization (ESI) source in both positive and negative ionization modes. We performed accurate quantitative analysis of 306 metabolites (including 60 amino acids, 55 fatty acids, 41 organic acids, 39 bile acids, 25 carbohydrates, 21 benzenoids, 20 carnitines, 9 indoles, 3 nucleosides, 9 phenylpropanoic acids and 16 others) in this study.

#### Data processing and analysis

The targeted raw data were processed through calibration curve of standards. Then, the raw data were analyzed by the iMAP software (Metabo-profile, Shanghai, China). Metabolites selected as biomarker candidates for further statistical analysis were identified on the basis of variable importance in the projection (VIP) threshold of 1.0 from the tenfold cross-validated OPLS-DA model, which was validated at a univariate level with p value < 0.05.

More detailed process descriptions of proteomics and metabolomics are provided in Additional file 1.

### Network pharmacological analysis

#### Data collection

For each of the two TCM Syndrome of CHD, we selected 3 formulae applied in clinic for the treatment of this Syndrome. The treatment of each formula to corresponding Syndrome was recommended in the Expert Consensus for Chinese Medicine Diagnosis and Treatment of Stable Angina Pectoris of CHD [14] and also recorded in the Chinese Pharmacopeia [15]. Active compounds of each herb in the formulae and their corresponding targets were collected from the Encyclopedia of Traditional Chinese Medicine (ETCM) [16], Traditional Chinese Medicine Systems Pharmacology Database and Analysis Platform (TCMSP) [17], and the high-throughput experiment- and reference-guided database of traditional Chinese medicine (HERB) [18]. Diseases highly related with coronary heart disease (CHD) were collected from DisGeNet database [19]. Drug classes for CHD related diseases and their Anatomical Therapeutic Chemical (ATC) codes, as well as drugs in these classes, were collected from DrugBank database [20].

#### Network construction and analysis

Protein–protein interaction networks were construction by GeneMania platform [21]. Metabolite-gene association networks were constructed by the “Network Analysis” module of MetaboAnalyst platform [22]. All networks were visualized and analyzed by Cytoscape software [23].

#### Functional annotation analysis

Functional annotation analysis for genes was conducted by DAVID [24] and STRING platform [25]. Functional annotation analysis for metabolites and genes was performed by “Pathway Analysis” and “Joint-Pathway Analysis” module of MetaboAnalyst platform.

## Results

### Characteristics of CHD patients with either CCQS or QSBS Syndrome

A total of 111 participants, including CHD patients with CCQS Syndrome (*n* = 44), QSBS (*n* = 37) and healthy controls (HC, *n* = 30) were enrolled at the Cardiology Clinic of Dongzhimen Hospital affiliated to Beijing University of Chinese Medicine or the surrounding communities in Beijing between August 2018 and December 2019. The demographic and clinical biochemical indicators of the participants were listed in Table 1. There were no significant differences in age, sex, BMI and laboratory data between CCQS and QSBS group. However, there were some disparities in the comorbidity of the two groups. CCQS group included higher percentage of patients with diabetes mellitus, whereas QSBS group contained higher percentage of patients with hypertension, hyperlipidemia, and cerebrovascular disease. The differences may reflect the respective characteristics of the two Syndromes to some extent.

**Table 1:** The demographic and clinical biochemical indicators of participants

	HC (n = 30)	CCQS (n = 44)	QSBS (n = 37)	p values
Basic information				
Age (year)	62.8 ± 9.4	67.2 ± 7.7	67.5 ± 7.8	0.048
Female	17 (56.7)	25 (56.8)	21 (56.8)	1.000
BMI (kg/m2)	24.7 ± 2.9	24.1 ± 3.9	25.2 ± 3.3	0.367
Arrhythmia	6 (20)	6 (13.6)	6 (16.2)	0.768
Diabetes mellitus	1 (3.3)	20 (45.5)	15 (40.5)	< 0.001
Hypertension	5 (16.7)	28 (63.6)	28 (75.7)	< 0.001
Hyperlipidemia	4 (13.3)	26 (59.1)	24 (64.9)	< 0.001
Cerebrovascular disease	2 (6.7)	4 (9.1)	15 (40.5)	< 0.001
Laboratory data				
TC (mmol/L)	5.2 ± 1.0	4.4 ± 1	4.3 ± 0.9	0.001
TG (mmol/L)	1.4 ± 0.9	1.5 ± 0.8	1.6 ± 0.7	0.704
HDL (mmol/L)	1.5 ± 0.3	1.2 ± 0.2	1.3 ± 0.3	0.005
LDL (mmol/L)	3.1 ± 0.8	2.6 ± 0.8	2.5 ± 0.7	0.002
Albumin (g/L)	44.0 ± 2.1	44.1 ± 2.7	45 ± 5.9	0.532
ALP (U/L)	89.9 ± 24.7	87.8 ± 20	86.4 ± 31.6	0.871
ALT (U/L)	20.4 ± 10.9	22.2 ± 12	21.3 ± 10.5	0.800
AST (U/L)	24.1 ± 5.1	24.5 ± 8.3	23.1 ± 6.1	0.665
Creatinine (µmol/L)	61.8 ± 11.3	65 ± 16.8	63.6 ± 16.5	0.694
GGT (U/L)	25.6 ± 16.9	29.5 ± 24.7	29.2 ± 17.4	0.717
Glu (mmol/L)	5.8 ± 1.2	6.8 ± 1.9	6.6 ± 1.7	0.03
HCY (µmol/L)	17.3 ± 13.5	14.9 ± 5.7	14.6 ± 5.3	0.377
hs-CRP (mg/L)	1.9 ± 2.1	2 ± 2.2	1.6 ± 1.9	0.666
Total protein (g/L)	74.4 ± 4.6	73.8 ± 4.7	73.4 ± 6.2	0.778
Urea (mmol/L)	5.2 ± 1.0	5.5 ± 2	5.9 ± 1.9	0.315
Uric acid (µmol/L)	305 ± 80.2	318.4 ± 79.9	344.3 ± 81	0.131

Values were mean ± SD or %; p values determined by one-way ANOVA and χ^2^ test

It is noted that the levels of TC and LDL in blood samples from healthy controls were higher than CHD patients, whereas high levels of LDL and TC have been known to be associated to CHD. However, these levels of most healthy controls were in the normal range, that is, LDL < 3.62 mmol/L (SUR method), and TG < 1.7 mmol/L (GPO-POD method). This bias could be due to the higher age of participants (most between 60 and 70), meanwhile participants in CHD group may have been given some lipid-lowering interventions.

### Proteomics profiling on CHD patients with either CCQS or QSBS syndrome

**Fig. 2:**
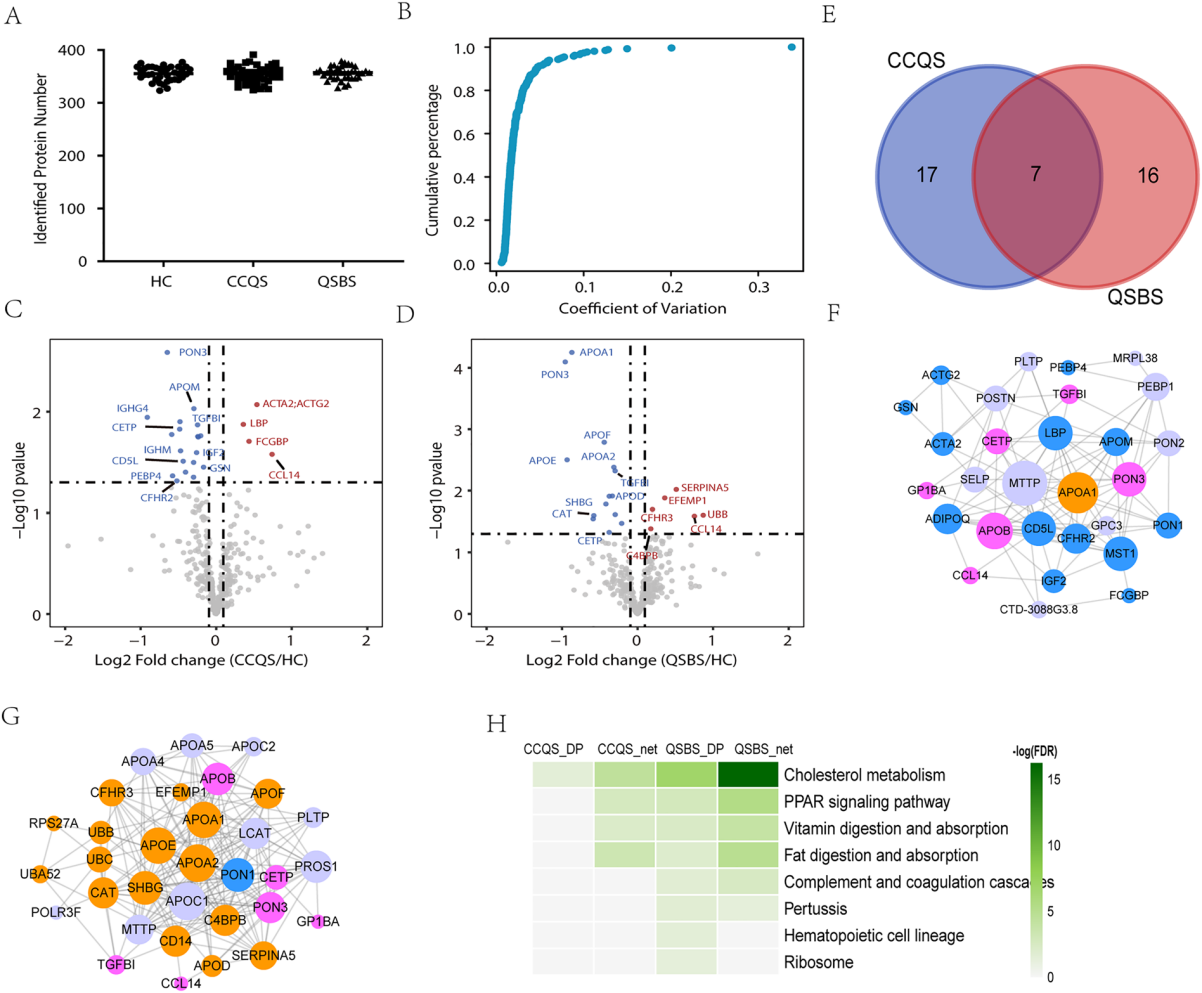
Analysis of the differential proteins (DPs) for the two classes of Syndromes. (**A)** The identified protein number in each group. (**B)** Coefficient of variation of proteins in Standard samples. The volcano plots of DPs between CCQS and HC group (**C**) or QSBS and HC group (**D**). (**E)** Overlaps between DPs of the two Syndromes. (**F**) Protein–protein interaction network for DPs of CCQS Syndrome. (**G)** Protein–protein interaction network for DPs of QSBS Syndrome. (**H**) KEGG pathways enriched with DPs and proteins in PPI networks for DPs of the two Syndromes. In **F**, **G**, Pink circles are common DPs of the two Syndromes, blue and orange circles are DPs specific for CCQS and QSBS Syndrome, respectively. Purple circles are proteins functionally associated with the DPs

In order to study the proteomic characteristics, we used the data independent acquisition (DIA) method to analyze the serum samples. In total, 470 proteins were identified. Averagely, 353, 355 and 354 proteins were identified in CCQS, QSBS and HC group, respectively (Fig. 2A). We also detected protein intensities covering nearly 6 orders of magnitude, with serum albumin being the most abundant one and macrophage receptor MARCO the lowest one (Additional file 1: Fig. S1A). Then correlation analysis was performed between samples, in which the correlation coefficients between each two samples were higher than 0.87, indicating high correlation (Additional file 1: Fig. S1B). Meanwhile, more than 95% of the proteins quantified in standard samples had coefficient of variation (CV) below 10% (Fig. 2B).

Next, to figure out the proteomic profile differences between CCQS and QSBS Syndrome, 388 proteins (missing values filled by R software) were included in subsequent analysis. Differential proteins (DPs) between CCQS and HC groups, as well as QSBS and HC groups (FC > 1.1, Student’s t-test, p-value < 0.05) were determined. There were 24 differential proteins between CCQS and HC groups, with 5 up-regulated and 19 down-regulated (Fig. 2C). A total of 23 differential proteins were determined between QSBS and HC groups, with 9 up-regulated and 14 down-regulated (Fig. 2D). By intersection analysis, 7 proteins were common DPs of the two Syndromes, while 17 and 16 proteins were specific DPs for CCQS and QSBS Syndrome, respectively (Fig. 2E).

**Fig. 3:**
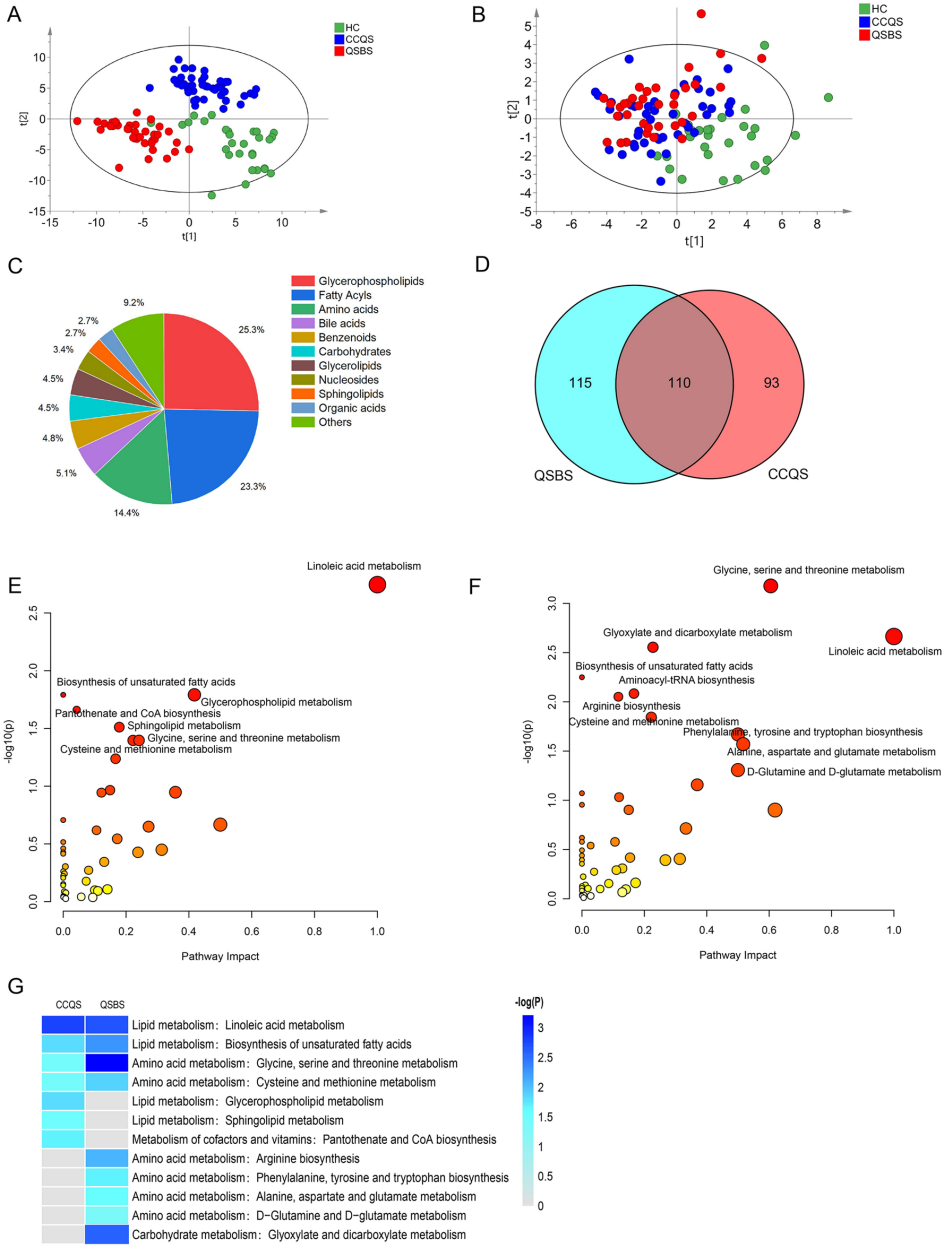
Metabolic analysis of CHD patients with two different Syndromes. (**A**, **B)** PLS-DA score plot of untargeted and targeted metabolome for two Syndromes. (**C)** The categories and proportions of DMs obtained from the two Syndromes. (**D)** Venn plot of the DMs filtered by OPLS-DA model and univariate analysis. (**E)** Differential metabolic pathways of DMs enrichment in CCQS Syndrome. (**F)** Differential metabolic pathways of DMs enrichment in QSBS Syndrome. (**G)** KEGG metabolic pathways enriched with DMs of the two Syndromes

We input DPs of the two Syndromes to GeneMania platform [21] to construct their protein–protein interaction (PPI) network, respectively. This platform uses the label propagation algorithm to score each gene in the entire human genomic-scale PPI network according to its links to all genes in the input list. Genes with higher scores are functionally associated with the input genes at higher extent, hence they are more likely to be affected by the input genes. For each of the input lists, we added 10 other genes with the top-ranking scores to construct the PPI network (Fig. 2F, G). Then, we used STRING platform [25] to perform KEGG pathway [26] enrichment analysis for the DPs and genes in the two PPI networks, respectively. Figure 3H showed that all the 4 groups of genes were enriched in the pathway of cholesterol metabolism, while 3 groups of genes were also enriched in PPAR signaling pathway, vitamin digestion and absorption, and fat digestion and absorption. This suggested that all the 4 pathways were associated with the occurrence and development of both CCQS and QSBS Syndrome. On the other hand, only the two groups of genes for QSBS Syndrome were enriched in 4 other pathways, including the pathway of complementary and coagulation cascades.

At last, we applied the Functional Annotation Clustering tool of DAVID to identify disease groups enriched with DPs of CCQS and QSBS (classification stringency was set as “High”.), respectively. Table 2 showed that DPs of both Syndromes were significantly enriched with genes involved in diseases directly related to CHD, including inflammation, cardiovascular diseases, coronary artery disease, and coronary atherosclerosis. Except these common diseases, DPs of CCQS were also enriched with disease genes of insulin resistance, metabolic Syndrome, and type 2 diabetes, while those of QSBS were enriched with genes associated with venous thromboembolism, brain ischemia, stroke, and Alzheimer's disease. The distinction of DP-enriched diseases was consistent with the comorbidity difference of clinic samples for the two Syndromes.

**Table 2:** Disease clusters enriched with differential proteins of the two Syndromes

Syndrome	Enrichment score/Rank	Disease term	#Mapped genes	Total genes	Percentage	P
CCQS	3.69/1	Myocardial infarct	5	121	20.83%	2.51E−05
		Coronary Artery Disease|	4	52	16.67%	5.63E−05
	3.39/2	Coronary Disease|Coronary heart disease| Inflammation|*Insulin Resistance*	5	59	20.83%	1.42E−06
		Atherosclerosis, coronary	5	155	20.83%	6.61E−05
		Cardiovascular disease	4	59	16.67%	8.23E−05
		*Metabolic syndrome*	4	165	16.67%	0.00168
		*Obesity*	5	369	20.83%	0.00177
		Cardiovascular Diseases	4	189	16.67%	0.00248
		*Diabetes, type 2*	5	439	20.83%	0.00334
QSBS	5.26/1	Myocardial ischemia	5	18	21.74%	7.85E−09
		Carotid atherosclerosis	5	21	21.74%	1.53E−08
		Atherosclerosis, coronary	6	155	26.09%	1.73E−06
		Recurrence|*Venous Thromboembolism*	4	58	17.39%	6.60E−05
		*Brain Ischemia*|Hypertension|Osteoporosis|*Stroke*	4	64	17.39%	8.87E−05
		Cardiovascular Diseases	4	67	17.39%	1.02E−04
		*Alzheimer's disease*	5	950	21.74%	0.03828

### Metabolomics profiling on CHD patients with either CCQS or QSBS syndromes

#### Syndrome-specific metabolic characters in CHD patients

To explore the metabolic differences between CCQS and QSBS Syndromes, we constructed a partial least squares discriminant analysis (PLS-DA) model. The serum metabolome of CCQS and QSBS patients were clearly separated from health control on the PLS-DA score plot in both untargeted (Fig. 3A) and targeted metabolomics (Fig. 3B). The identified differential metabolites (DMs) belonged to 10 categories, in which glycerophospholipids (25.3%), fatty acyls (23.3%) and amino acids (14.4%) were the three most influential metabolites (Fig. 3C). The 200 metabolites were significantly changed in serum of CCQS patients and the 225 metabolites were significantly changed in QSBS patients, including commonly altered 110 DMs in two Syndromes (Fig. 3D and Additional file 1: Table S1). These results suggested the Syndrome-specific metabolic characters in CHD patients, as well as common metabolic characters between CCQS and QSBS Syndromes.

#### Syndrome-specific metabolic pathways in CHD patients

On the basis of identified DMs, a total of 91 and 97 DMs with KEGG IDs were further analyzed, which were significantly altered in CHD patients of either CCQS or QSBS Syndrome, respectively. Then, we used MetaboAnalyst platform to perform functional enrichment analysis for the DMs. Compared with healthy controls, seven differential metabolic pathways were enriched in the serum of CCQS patients, including lipid metabolism (biosynthesis of unsaturated fatty acids, glycerophospholipid metabolism, linoleic acid metabolism, sphingolipid metabolism), amino acid metabolism (cysteine and methionine metabolism, glycine, serine and threonine metabolism), metabolism of cofactors and vitamins (pantothenate and CoA biosynthesis) (Fig. 3E). Compared with healthy controls, ten differential metabolic pathways were enriched in the serum of QSBS patients, including lipid metabolism (linoleic acid metabolism and biosynthesis of unsaturated fatty acids), amino acid metabolism (alanine, aspartate and glutamate metabolism, cysteine and methionine metabolism, glycine, serine and threonine metabolism, and arginine biosynthesis), metabolism of other amino acids (D-glutamine and D-glutamate metabolism), translation (aminoacyl-tRNA biosynthesis), carbohydrate metabolism (glyoxylate and dicarboxylate metabolism) (Fig. 3F).

Figure 3G showed that all the 2 groups of metabolites were enriched in 2 pathways of lipid metabolism (linoleic acid metabolism, and biosynthesis of unsaturated fatty acids), as well as 2 pathways of amino acid metabolism (glycine, serine and threonine metabolism; cysteine and methionine metabolism). In addition, DMs of CCQS were enriched in 2 more pathways of lipid metabolism, *i.e.*, glycerophospholipid metabolism, and sphingolipid metabolism; while DMs of QSBS were enriched in 4 pathways of amino acid metabolism, including arginine metabolism, tyrosine metabolism, and two pathways of glutamate metabolism.

**Fig. 4:**
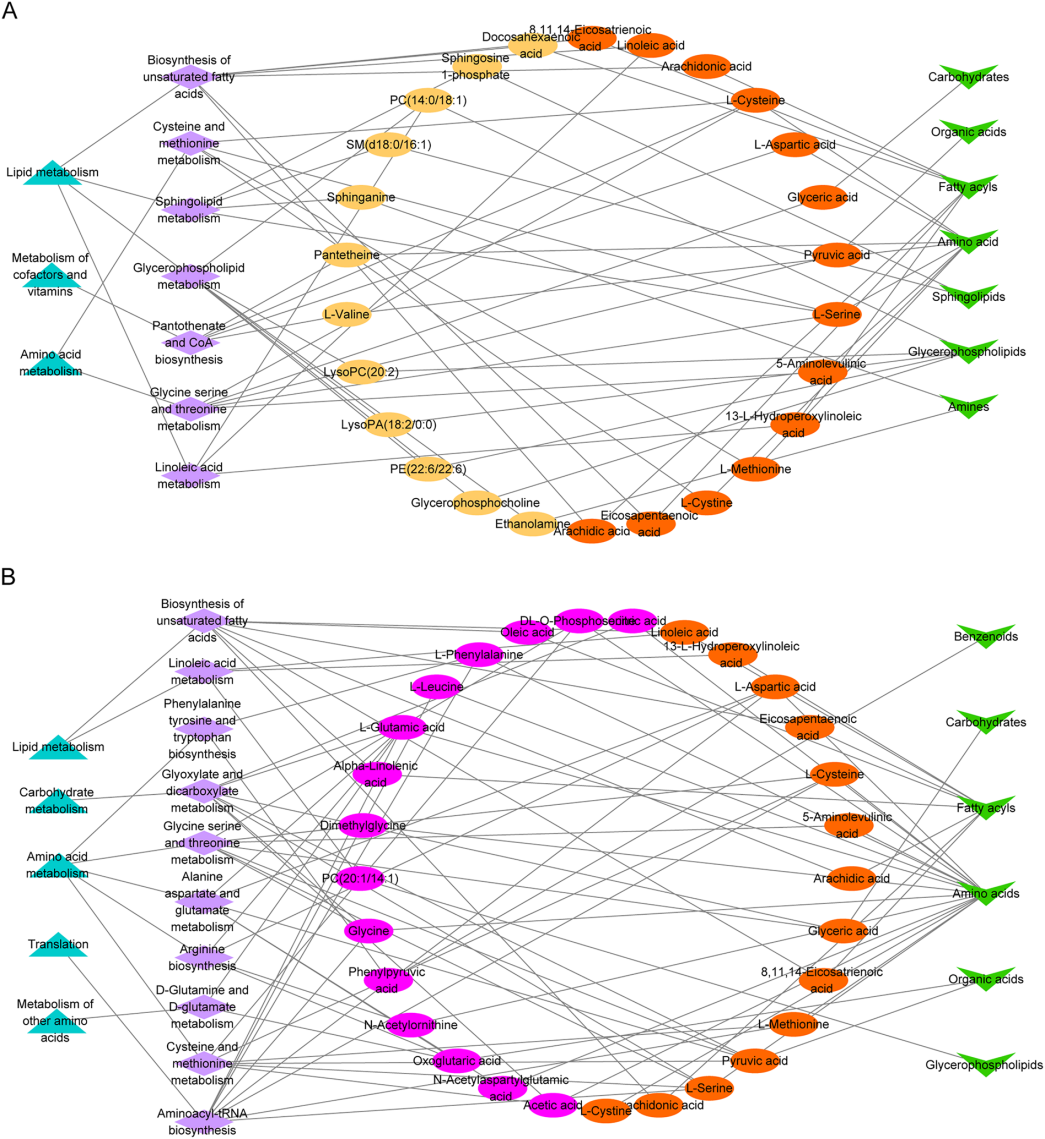
Differential metabolic network analysis of DMs and diagnostic analysis of metabolic biomarkers panel. (**A)** Differential metabolic network analysis of DMs enrichment in Syndrome of CCQS. (**B)** Differential metabolic network analysis of DMs enrichment in Syndrome of QSBS

Next, we mapped the network of differential metabolic pathways (p < 0.05) enriched with DMs through Cytoscape software. As shown in Fig. 4A, B, the 12 DMs, including 5 glycerophospholipids (PC (14:0/18:1), lysoPC (20:2), lysoPA (18:2/0:0), PE (22:6/22:6) and glycerophosphocholine), 2 sphingolipids (sphingosine 1-phosphate and SM (d18:0/16:1)), 1 fatty acyls (docosahexaenoic acid), 2 amino acid (l-valine and pantetheine), 2 amines (sphinganine and ethanolamine), were unique metabolites in the serum of patients with CCQS. The 15 DMs, including 10 amino acids (DL-O-phosphoserine, l-phenylalanine, glycine, isocitric acid, dimethylglycine, N-acetylornithine, pyroglutamic acid, l-glutamic acid, l-leucine and acetic acid), 2 fatty acyls (oleic acid and alpha-linolenic acid), 1 glycerophospholipids (PC (20:1/14:1)), 1 benzenoids (phenylpyruvic acid), and 1 organic acid (oxoglutaric acid), were unique metabolites in the serum of patients with QSBS.

### Metabolome-proteome integrated analysis and ROC diagnosis

**Fig.5:**
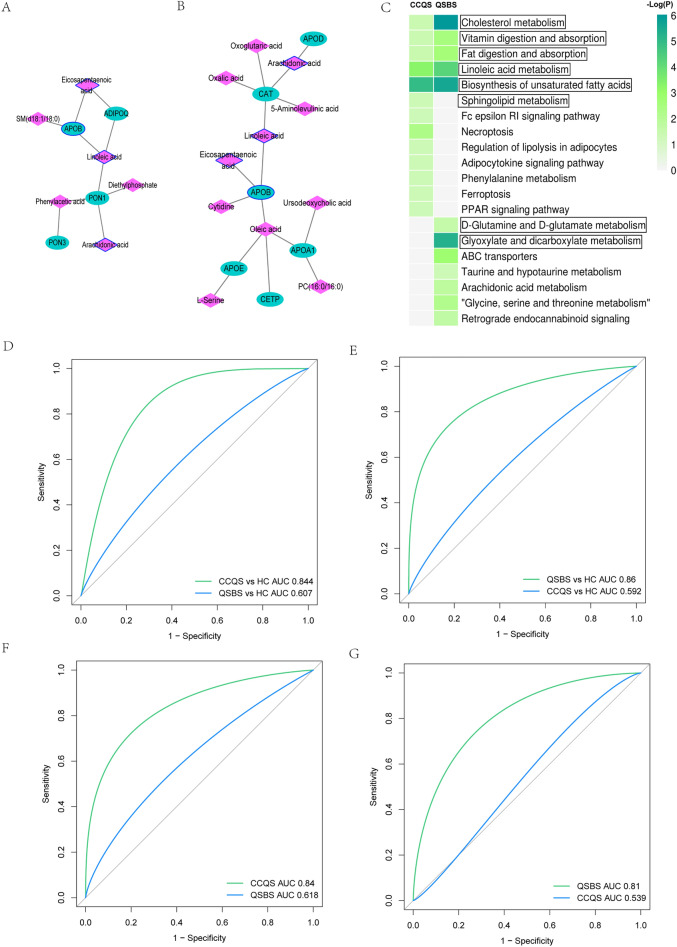
Metabolome-proteome integrated analysis and ROC diagnosis. (**A)** Metabolome-proteome integrated analysis of CCQS patients. (**B)** Metabolome-proteome integrated analysis of QSBS patients. (**C)** KEGG metabolic pathways enriched with DMs and DPs of the two Syndromes. Pathways within the rectangles were also identified only by metabolomics or proteomics data. (**D)** The ROC diagnostic analysis of biomarker panel (ACTA2, FCGBP, LBP, IGHM) for different Syndromes. (**E)** The ROC diagnostic analysis of biomarker panel (CFHR3, EFEMP1, UBA52, APOD, CAT) for different Syndromes. (**F)** The ROC diagnostic analysis of biomarker panel (l-valine, pantetheine, lysoPC (20:2) and sphingosine 1-phosphate) for different Syndromes. (**G)** The ROC diagnostic analysis of biomarker panel (pyroglutamic acid, acetic acid, PC (20:1/14:1) and socitric acid)

First, we used MetaboAnalyst platform to study the interactions between metabolites and proteins associated with the same Syndrome. We input DMs and DPs of the same Syndrome into the “Network Analysis” module of MetaboAnalyst and constructed the integrated metabolite-protein network of Syndrome. As shown in Fig. 5A, B, the two Syndromes have different metabolite-protein networks. Six metabolites (including diethylphosphate, phenylacetic acid, SM(d18:1/18:0), arachidonic acid, eicosapentaenoic acid and linoleic acid) and 4 proteins (including APOB, PON1, PON3 and ADIPOQ) form the CCQS network, 11 metabolites (including ursodeoxycholic acid, cytidine, eicosapentaenoic acid, oleic acid, l-serine, 5-aminolevulinic acid, arachidonic acid, linoleic acid, PC(16:0/16:0), oxalic acid, oxoglutaric acid) and 6 proteins (including APOA1, APOE, APOB, CETP, CAT and APOD) form the QSBS network. Three fatty acids metabolites (arachidonic acid, linoleic acid, eicosapentaenoic acid) and 1 protein (APOB) are the same metabolites and protein included in the two networks. Arachidonic acid, linoleic acid, eicosapentaenoic acid are fatty acids metabolites significantly associated with inflammation, which suggests that inflammation may be a common feature of the two Syndromes. Figure 5C showed pathways enriched with DMs and DPs of the two Syndromes (p < 0.05). All the 5 common pathways were also identified only by proteomics analysis (cholesterol metabolism, vitamin digestion and absorption, fat digestion and absorption) or metabolomics analysis (linoleic acid metabolism, biosynthesis of unsaturated fatty acids). One pathway specific for CCQS (sphingolipid metabolism) were also identified by metabolomics analysis. Two pathways specific for QSBS (D-glutamine and D-glutamate metabolism; glyoxylate and dicarboxylate metabolism) were also identified only by metabolomics analysis.

Further, we performed ROC diagnostic analysis on the unique DPs and DMs of the two Syndromes. A binary logical regression analysis was carried out for the CCQS and QSBS based DPs and DMs to produce a biomarker panel. In the proteomics experiment, we found that a biomarker panel (ACTA2, FCGBP, LBP, IGHM) could accurately distinguish CCQS patients from healthy controls. as indicated by the area under the receiver operating curve (AUC), which had a value up to 0.844 (Fig. 5D). However, we found that the biomarker panel obtained poor performance when discriminating between QSBS and healthy controls due to decreased specificity and sensitivity (AUC = 0.607) (Fig. 5D). Similarly, a biomarker panel (CFHR3, EFEMP1, UBA52, APOD, CAT), could accurately distinguish QSBS patients from healthy controls (AUC = 0.86) and obtained poor performance when discriminating between CCQS and healthy controls due to decreased specificity and sensitivity (AUC = 0.592) (Fig. 5E). In the metabolomics experiment, we also found that two biomarker panel (l-valine, pantetheine, lysoPC (20:2), sphingosine 1-phosphate) and (pyroglutamic acid, acetic acid, PC (20:1/14:1), socitric acid), could accurately distinguish CCQS and QSBS patients from healthy controls, AUC were 0.84 and 0.81, respectively (Fig. 5F, G). The biomarker panel obtained poor performance when discriminating between another Syndrome and healthy controls due to decreased specificity and sensitivity (Fig. 5F, G).

### Network pharmacology analysis on TCM formulae used in the treatment of CHD patients with either CCQS or QSBS syndrome in clinic

To investigate features of the two distinct Syndromes from TCM formulae specifically used to treat them, we selected 6 TCM formulae recorded in the Chinese Pharmacopeia and extensively used for the treatment of the two TCM Syndromes of CHD in clinic. Each Syndrome corresponded to 3 formulae. The formulae prescribed for CCQS Syndrome were Shexiang Baoxin Pill, Guanxin Suhe Pill, and Kuanxiong Aerosol; while those for QSBS Syndrome were Suxiao Jiuxin Pill, Xuefu Zhuyu Pill, and Compound Danshen Drop Pill (Table 3). For simplicity, we coded the formulae corresponding to Syndrome CCQS and QSBS as F1 and F2, respectively; and the three formulae of F1 type were coded as F1-1, F1-2 and F1-3; the formulae of F2 type were coded as F2-1, F2-2 and F2-3 (Table 3; See Additional file 1: Table S2 for herbs in each formula).

**Table 3:** The number of herbs, potential bioactive compounds and corresponding putative targets for each TCM formula

Syndrome/Formula code	TCM formula	Formula code	# Herbs	#Active compounds	# Active compounds with targets	#Putative targets
CCQS/F1	Shexiang Baoxin Pill	F1-1	7	99	74	702
	Guanxin Suhe Pill	F1-2	5	58	36	280
	Kuanxiong Aerosol	F1-3	5	113	81	368
QSBS/F2	Suxiao Jiuxin Pill	F2-1	2	63	45	301
	Xuefu Zhuyu Pill	F2-2	11	397	312	822
	Compound Danshen Drop Pill	F2-3	3	137	111	647
Total			26	696	528	1246

For each herb in the TCM formulae, we first collected its chemical ingredients and corresponding putative targets from ETCM [16] and TCMSP [17] database. We filtered active compounds by OB $\ge 30\%$ and DL $\ge 0.18$ for ingredients from TCMSP database, while ingredients from ETCM database whose Druglikeness Gradings were good or moderate were considered as active compounds. Then, from the Chinese Pharmacopeia we searched quality markers (Q-markers) of each herb, whose pharmacological effects have been validated; we then searched their corresponding targets of the Q-markers from the HERB database [18]. We combined all data and finally obtained 696 active compounds for the 26 distinct herbs in the 6 formulae (Additional file 1: Table S3), in which 528 active compounds had 1246 putative targets (Table 3 and Additional file 1: Table S4).

**Fig. 6:**
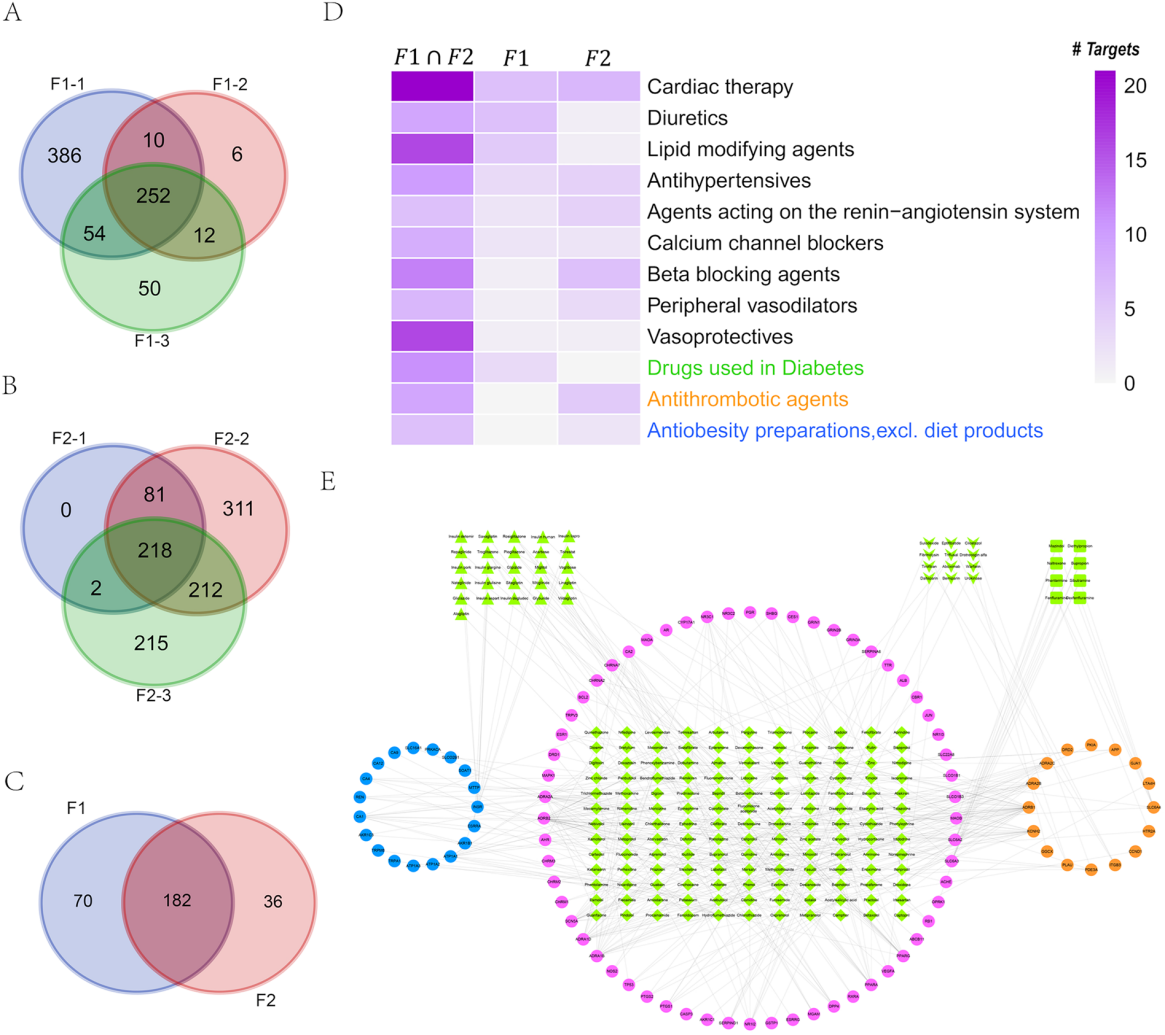
Analysis of the signature targets for the two classes of formulae. (**A)** Overlaps between the targets of the 3 formulae for CCQS. (**B)** Overlaps between the targets of the 3 formulae for QSBS. (**C)** Overlaps between the signature targets of the two classes of formulae. (**D)** Number of signature targets that overlapped with targets of FDA-approved CHD-related drugs, where black, green, orange, and blue text were drug categories for cardiovascular system (ATC ID:C), diabetes (ATC ID: A10), thrombotic events (ATC ID: B01), and obesity (ATC ID: A08), respectively. (**E)** Target-drug network between signature targets and FDA-approved CHD-related drugs. Pink cercles were common signatures for the two classes of formulae, while blue and orange circles were F1- and F2-specific signature targets, respectively. Diamond, triangle, V-type, and rectangle nodes were drugs for cardiovascular system, diabetes, ischemic stroke, and obesity, respectively

We considered the common targets of the 3 formulae for CCQS and QSBS Syndrome as signature targets of the corresponding class of formulae, respectively. There were 252 and 218 signature targets for the two classes of formulae, respectively (Fig. 6A, B). The two classes of formulae had 182 common signature targets, we considered the 70 and 36 ones which were signature targets of only F1 or F2 formulae as F1- and F2-specific signature targets of the two classes of formulae, respectively (Fig. 6C).

To understand the effects of the two classes of formulae, we compared the signature targets with targets of FDA-approved CHD-related drugs. We first searched DisGenet database [19] with key word “Coronary Heart Disease” for CHD-related diseases. Setting threshold as JIg > 0.2 and JIv > 0.05, where Jig and JIv denoted Jaccard index based on shared genes and shared variants, respectively, we identified 15 diseases highly associated with CHD (Additional file 1: Table S5). In DrugBank database [20], drugs treating these diseases could be classified to 4 categories according to their Anatomical Therapeutic Chemical (ATC) codes, i.e., drugs for cardiovascular system (ATC ID: C), diabetes (ATC ID: A10), obesity (ATC ID: A08), and ischemic stroke (ATC ID: B01). 

Then we mapped the common and specific signature targets to targets of the 4 categories of drugs in DrugBank database. Note that here we only studied pharmacological targets in DrugBank and excluded transporters, carriers, and enzymes of the drugs. As shown in Fig. 6D and E, the common feature targets had overlaps with drug targets of all the 4 categories. Both F1- and F2-specific feature targets overlapped with targets of drugs for cardiovascular system. However, F1-specific feature targets also overlapped with drug targets of diabetes, while F2-specific feature targets had intersection with drug targets for ischemic stroke and obesity. This result suggested that, besides treatment to CHD, F1 formulae paid more attention in the treatment of diabetes complications of CHD, while F2 formulae had higher efficacy to treat ischemic stroke complications of CHD. The differences in specific feature targets were consistent with the disparities of disease comorbidity in clinic samples, indicating that the targets and pathways regulated by formulae specific for a Syndrome could be the Syndrome-specific dysfunction objects.

### Integrated analysis between network pharmacology and multi-omics results

Our network pharmacological analysis suggested that targets of TCM formulae specific for a Syndrome could be dysfunction genes in the development of the Syndrome. Advances in technology have made it increasingly possible to study multiple biological aspects from genomes, proteomes and metabolomics to phenotypic analysis. Integrative analyses that use information across these data modalities promise to deliver more comprehensive insights into the biological systems under study [27, 28]. Thus we conducted integrated analysis of network pharmacology, proteomics, and metabolomics in this section.

**Fig. 7:**
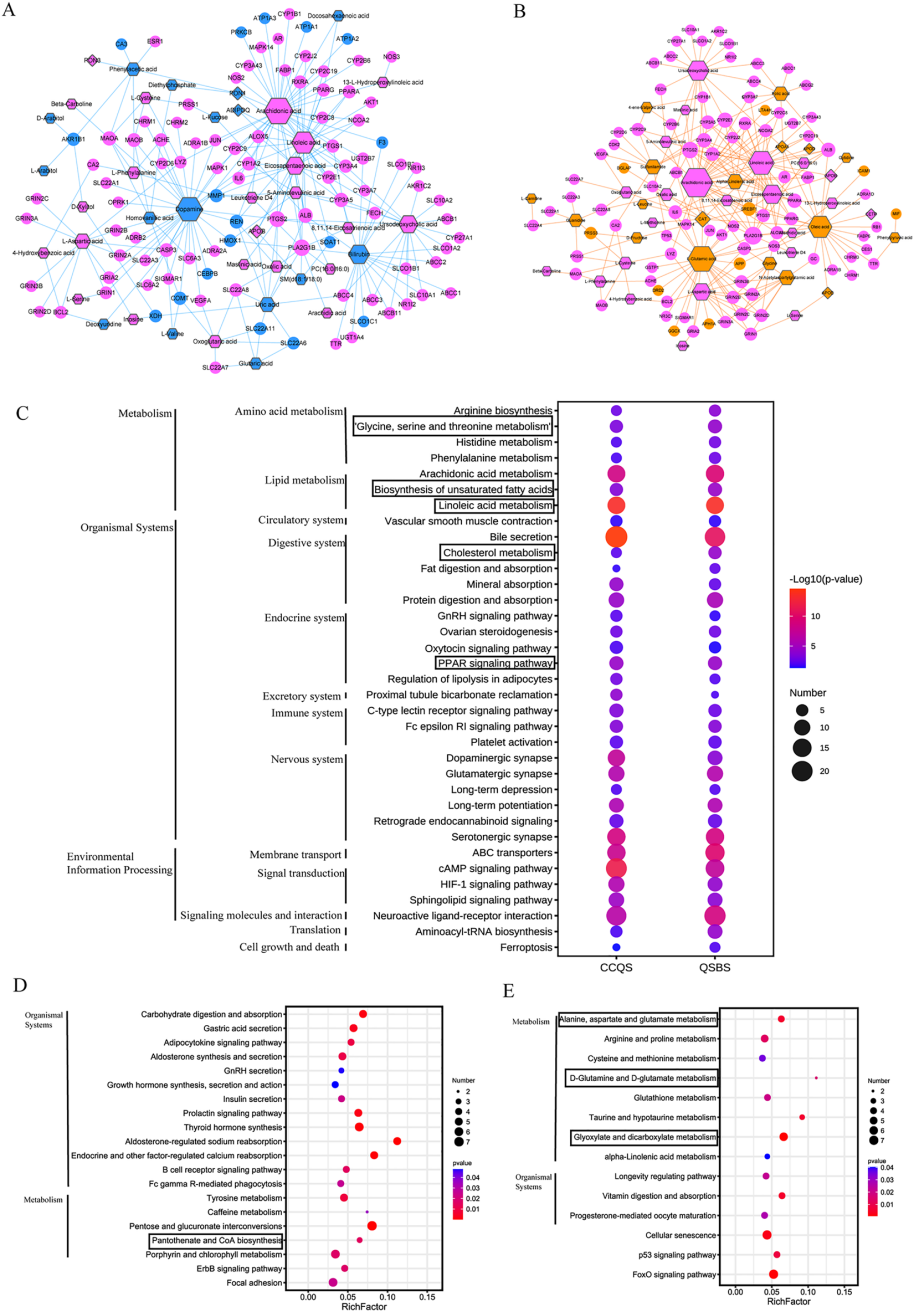
Integrated network analysis of multi-omics data. Integrated metabolite-gene network for CCQS (**A**) and QSBS Syndrome (**B**). Circles are feature targets of TCM formulae, diamonds are DPs, hexagons are metabolites. Pink nodes are molecules related to both Syndromes, blue and orange nodes are molecules related to CCQS and QSBS, respectively. (**C)** Common pathways enriched with molecules in integrated metabolite-gene networks of CCQS and QSBS. (**D)** Pathways only enriched with molecules in integrated metabolite-gene networks of CCQS. (**E)** Pathways only enriched with molecules in integrated metabolite-gene networks of QSBS. Pathways within the rectangles were also identified only by metabolomics or proteomics data

We used MetaboAnalyst platform to study the interactions between metabolites and genes associated with the same Syndrome. We input DMs, DPs, and feature targets of CCQS Syndrome into the “Network Analysis” module of MetaboAnalyst and constructed the integrated metabolite-gene network of CCQS (Fig. 7A). This network included interactions between 35 DMs and 102 genes related with CCQS Syndrome. Besides common molecules corresponding to the two Syndromes, this network contains 14 DMs and 2 DPs specific for CCQS Syndrome, and 17 F1-specific feature targets. The metabolite-gene network for QSBS was built similarly, which includes 14 DMs and 4 DPs specific for QSBS Syndrome and 10 F2-specific feature targets (Fig. 7B).

We then applied MetaboAnalyst platform for functional annotation of the two metabolite-gene networks. We focused the analysis on the basic biological pathways of KEGG database, which contains 5 sections: Metabolism, Genetic Information Processing, Environmental Information Processing, Cellular Processes, and Organismal Systems. All the molecules, including DMs, DPs and feature targets, in the metabolite-gene network of CCQS were input into the “Joint-Pathway Analysis” module of MetaboAnalyst and 86 pathways enriched with molecules related with CCQS were identified (p < 0.05). Similarly, 80 pathways were found to be enriched with molecules associated with QSBS (p < 0.05). We conducted intersection analysis for the two groups of pathways and discovered that the two Syndromes shared 66 pathways, in which the top 35 pathways were show in Fig. 7C. There were 20 and 14 pathways specific for CCQS and QSBS (Fig. 7D, E), respectively. Seven of the 66 common pathways were metabolic pathways, in which 3 were also identified only by metabolomics results, *i.e.*, glycine, serine and threonine metabolism; biosynthesis of unsaturated fatty acids; and linoleic acid metabolism. Two common pathways identified by proteomics analysis (cholesterol metabolism, PPAR signaling pathway) were also identified by integrated analysis. One pathway specific for CCQS (pantothenate and CoA biosynthesis) were identified both in metabolomics and integrated analysis. Three pathways specific for QSBS (D-Glutamine and D-glutamate metabolism; alanine, aspartate and glutamate metabolism; glyoxylate and dicarboxylate metabolism) identified by metabolomics analysis, were also identified here.

Next, we applied DAVID platform to identify disease groups enriched with Syndrome-specific genes included in the integrated networks. We input the 2 DPs and 17 feature targets specific for CCQS Syndrome in the metabolite-gene network into DAVID and performed functional annotation clustering with classification stringency “High”. QSBS-specific genes in the metabolite-gene network were analyzed in the same way. Table 4 showed that more than 20% specific genes of both Syndromes were significantly involved in coronary atherosclerosis and other diseases directedly related to CHD. Except these common diseases, CCQS-specific genes were also enriched with disease genes of metabolic Syndrome, while those of QSBS were enriched with genes associated with cerebrovascular disease, stroke, and hyperlipidemias. In addition, CCQS-specific DPs involved in these diseases were PON1 and ADIPOQ, while QSBS-specific DPs involved in these diseases were APOE and APOA1.

**Table 4:** Disease clusters enriched with Syndrome-specific genes in the metabolite-gene networks

Syndrome	Enrichment score/Rank	Disease Term	P value	Mapped genes
CCQS	3.09/1	*Metabolic syndrome*	5.28E−05	MMP1, *PON1*, *ADIPOQ*, HMOX1, F3
		Atherosclerosis, coronary	0.001006	MMP1, *PON1, ADIPOQ*, HMOX1
		Atherosclerosis	0.010393	MMP1, *PON1, ADIPOQ*, HMOX1
QSBS	2.58/1	*Hyperlipidemias*	7.44E−04	SREBF1, *APOA1, APOE*
		Hypercholesterolemia	9.77E−04	SREBF1, *APOA1, APOE*
		Plasma HDL cholesterol (HDL-C) levels	0.024904	SREBF1, *APOA1, APOE*
	2.41/2	*Cerebrovascular disease*; sickle cell anemia	2.12E−04	*APOA1, APOE*, ICAM1
		Coronary Artery Disease	0.001195	*APOA1, APOE*, ICAM1
		Acute Coronary Syndrome	0.002469	*APOA1, APOE*, ICAM1
		Hepatitis C|Remission, Spontaneous	0.005417	*APOA1, APOE*, ICAM1
		Atherosclerosis, coronary	0.010151	*APOA1, APOE*, ICAM1
		*Stroke*	0.100306	*APOA1, APOE*, ICAM1

Genes of Italic characters are DPs

## Discussion

In this study, we proposed a strategy which integrated clinic samples, multi-omics study, and network pharmacology to investigate the biomedical basis of two Syndromes (CCQS and QSBS) of CHD.

We collected clinic samples and conducted proteomic and metabolomic experiments using serum samples of the participants. Statistics suggested that clinic samples of the two Syndromes had no differences in age, sex, BMI and laboratory indexes, but the comorbidity features were different. CCQS group included higher percentage of patients with diabetes, whereas more patients in QSBS group had complications of cerebrovascular disease. Our functional annotation clustering for proteomics and metabolomics results also reflected the comorbidity difference in clinic samples of the two Syndromes. Furthermore, network pharmacological study on 6 TCM formulae used for the treatment of two Syndromes revealed that the 3 CCQS formulae paid more attention on the treatment for diabetes complication of CHD, while the 3 QSBS formulae focused more on the management of ischemic stroke complication related to CHD. This result suggested that the comorbidity discrepancy between the two Syndromes might be due to fundamental differences in the two Syndromes, rather than a deviation in sample collection. Further research and more evidence are needed for confirming this observation about comorbidity.

By analyzing differential proteins (DPs) of the two Syndromes identified from proteomic experiments of clinic serum samples, it was found that DPs of both Syndromes were enriched in the pathways of cholesterol metabolism, PPAR signaling, vitamin digestion and absorption, and fat digestion and absorption, suggesting that these 4 pathways were involved in the disease process of both Syndromes. It has been recognized for decades that CHD is strongly related with endogenous or exogenous lipids. Cholesterol is made by the body through the digestion and absorption of dietary fat, and plasma LDL cholesterol levels have an independent correlation to the risk of CHD [29]. PPARs (peroxisome proliferator-activated receptors) consist of three related transcription factors: PPARA, PPARD, and PPARG. They control the proliferation of peroxisomes, which are organelles involved in fatty acid metabolism. PPARs are critical regulators of lipid metabolism and play a role in biological processes associated with the onset and progression of CHD, such as fatty acid oxidation, insulin sensitivity, and inflammatory signaling [30]. On the other hand, only DPs of QSBS were enriched in the pathways of complementary and coagulation cascades. Coagulation cascades are a set of proenzyme-to-serine protease conversions that result in thrombin production. It has been suggested that abnormalities in the plasmatic coagulation system are linked to ischemic stroke [31]. This result indicated that the QSBS is more strongly associated with ischemic stroke.

From metabolomic results of clinic serum samples, it was found that differential metabolites (DMs) of both Syndromes were enriched in 4 pathways: linoleic acid metabolism; biosynthesis of unsaturated fatty acids; glycine, serine and threonine metabolism; and cysteine and methionine metabolism, suggesting that the 4 pathways were associated with both Syndromes of CHD. It has been known that dietary unsaturated fat intake is inversely correlated with CHD risk [32]. Linoleic acid is an unsaturated fatty acid that is necessary for the production of eicosanoids, which are significant regulators of platelet aggregation, blood pressure, and coronary flow [32]. Specific amino acids have been known to play important role in the etiology of CHD by influencing lipid metabolism. Several amino acids involved in our identified pathways, including glycine, cysteine, alanine, glutamate, and glutamine, were found to have a significant impact on macrophage atherogenicity, while macrophages initiate the atherogenesis process by accumulating high amounts of circulating lipids [33]. In addition, our study showed that DMs of CCQS were enriched in 2 more pathways of lipid metabolism (glycerophospholipid metabolism, and sphingolipid metabolism) and one pathway of cofactors and vitamins metabolism (pantothenate and CoA biosynthesis); while DMs of QSBS were enriched in 4 pathways of amino acid metabolism, including arginine metabolism, tyrosine metabolism, and two pathways of glutamate metabolism. Diabetes mellitus is the most common endogenous cause for disorder of lipid metabolism [34]. Glycerophospohlipids and sphingolipids were found to be considerably lower in T2B patients [35, 36]. Khan et al.observed that downregulated sphingolipid metabolism in mice reduced insulin sensitivity and caused dysfunction of pancreatic β cells [35]. The pancreatic β cells are endocrine cells for synthetizing, storing, and releasing insulin. The disruption of pancreatic β cell function was key factors for the incitation and progression of T2D [37]. Pantothenate is a necessary precursor for the formation of coenzyme A (CoA), a ubiquitous cofactor involved in a wide range of metabolic pathways. The control of CoA is critical for metabolic flexibility and glucose homeostasis [38]. Glutamate is the most abundant free amino acid in the central nervous system. The function of glial glutamate transporters is hindered in the pathological situation of ischemic stroke, resulting in excessive glutamate release from neurons and glial cells. This process triggers excitotoxicity which damages the surrounding nervous tissue and impairs normal brain functions [39]. Hence, the dysfunction of glutamate metabolism is highly associated with ischemic stroke. Therefore, the analysis of metabolic pathways enriched with the DMs suggested that CCQS and CSBS Syndromes were more strongly related with diabetes and ischemic stroke, respectively.

At last, by integrating data from network pharmacology, proteomics, and metabolomics study, we constructed integrated metabolite-gene network for the two Syndromes and conducted functional annotation for molecules in the two networks, respectively. The results of integrated analysis and omics (metabolomics and proteomics) analysis were consistency in 5 common pathways for two Syndromes (glycine, serine and threonine metabolism; biosynthesis of unsaturated fatty acids; linoleic acid metabolism; cholesterol metabolism; PPAR signaling pathway), 1 specific pathway for CCQS (pantothenate and CoA biosynthesis), and 3 specific pathways for QSBS (D-glutamine and D-glutamate metabolism; alanine, aspartate and glutamate metabolism; glyoxylate and dicarboxylate metabolism). Disease clustering analysis for Syndrome-specific genes in the integrated networks suggested that the genes of both Syndromes were enriched in coronary atherosclerosis. In addition, CCQS-specific and QSBS-specific genes were also enriched in metabolic Syndrome and cerebrovascular disease, respectively, in which Syndrome-specific DPs involved in the diseases were PON1 and ADIPOQ for CCQS, APOE and APOA1 for QSBS. According to our proteomics experiments, the abundance of PON1 and ADIPOQ were significantly lower in CCQS patients compared with healthy control, while both APOE and APOA1 were down-expressed in QSBS patients compared with healthy control, suggesting that the two pairs of DPs could play important roles in the pathology of the two Syndromes.

PON1 (serum paraoxonase/arylesterase 1) is one of the 3 members of the paraoxonase family, any of which can degrade lipid peroxides in HDL and LDL [40]. Primarily expressed in the liver and then secreted into the bloodstream, PON1 was shown mainly bound to HDL in blood [41] and inhibited macrophage cholesterol biosynthesis [42]. Low PON1 levels were closely linked to the start and progression of atherosclerosis [43]. PON1 also has the function of regulating fasting blood glucose levels, glucose tolerance, and insulin sensitivity [44]. Previous studies have found lower PON1 activity in both T1DM and T2DM [43].

ADIPOQ (adiponectin) is an adipokine produced by adipose tissue that regulates lipid metabolism and insulin sensitivity. In vitro experiments have shown that ADIPOQ suppressed the expression of endothelial adhesion molecules, the production of TNFa and IL6, the proliferation of vascular smooth muscle cells, and the transformation of macrophage to foam cells [45, 46]. Lower plasma concentrations of adiponectin were found associated with coronary artery disease [47]. ADIPOQ could increase insulin sensitivity [48] and its circulating levels have been found inversely associated with the risk of type 2 diabetes [49].

APOE (apolipoprotein E) is an apolipoprotein important for the transport and metabolism of lipids. It is abundant in the brain [50]. APOE could clear cholesterol-rich lipoproteins from plasma, enhance the release of cellular lipid from macrophage foam cells, suppresses the migration and proliferation of vascular smooth muscle cells, and reduce lipid oxidation [51–53]. ApoE is also involved in the activation of endothelial cells and platelets, as well as the phagocytotic clearance of apoptotic materials [54, 55]. The expression of APOE is associated with the pathology of atherosclerosis and ischemic stroke [51, 56].

APOA1 (apolipoprotein A-I) is the major protein component of high-density lipoprotein (HDL) complexes. By boosting cholesterol efflux from tissues, it plays a key function in the reverse transfer of cholesterol from tissues to the liver for excretion. The quantity of ApoA1 in plasma has been found to be substantially associated with the level of HDL cholesterol in the blood [57]. According to epidemiological research, the ratio of APOA1 to APOB was a good predictor of coronary heart disease risk [57]. Lower levels of APOA1 were found to be correlated with an increased risk of the acute onset of ischemic stroke [58].

To sum up, based on clinic samples and collected data, our study revealed the differences between the two Syndromes from three aspects: biological processes, comorbidities, and potential biomarkers. We should point out some limitations in this research. The first is that the size of clinic samples is relatively small due to the limitation of time period, as well as strict inclusion and exclusion criteria. Second, formulae’s ingredients and targets are collected from databases. Although we integrated our data from most TCM databases currently available in public and studied multiple TCM formulae to enhance the reliability of targets, the inherent quality of the databases themselves may cause some deviation in our results.

## Conclusion

By integrating clinic samples, multi-omics study and network pharmacology, our study suggested that both CCQS and QSBS Syndrome of CHD were related with the dysfunction of 5 pathways: glycine, serine and threonine metabolism; biosynthesis of unsaturated fatty acids; linoleic acid metabolism; cholesterol metabolism; and PPAR signaling pathway. Moreover, CCQS Syndrome of CHD patients were specifically characterized with altered pantothenate and CoA biosynthesis, while disordered pathways of D-glutamine and D-glutamate metabolism; alanine, aspartate and glutamate metabolism, and glyoxylate and dicarboxylate metabolism were present in CHD patients with QSBS Syndrome. Furthermore, our results indicated that the down-expressed PON1 and ADIPOQ could related with the pathology of CCQS Syndrome, while down-expressed APOE and APOA1 for QSBS Syndrome. In addition, both clinic samples and network pharmacological results suggested that CCQS or QSBS Syndrome was highly related with the pathology of diabetes or ischemic stroke, respectively. Further research needs to be conducted to investigate whether such comorbidity phenomena is correlated with the inherent difference of the two Syndromes. This study helps us to understand the TCM Syndromes from perspective of modern biomedical science and facilitate the application of TCM theory in the practice of precision medicine.

## Supplementary Information

**Additional file 1.** Materials and methods for proteomics and metabolomics experiments. **Table S1.** List of differential metabolites. **Table S2.** Herbs in the 6 TCM formulae under study. **Table S3.** Active compounds in each formula. **Table S4.** Putative targets in each formula. **Table S5.** Diseases the most highly associated with CHD and corresponding drug ATC codes.

## Data Availability

The data used and/or investigated during the present study are available from the corresponding author upon reasonable request.

## Abbreviations

TCM: Traditional Chinese medicine
CHD: Coronary heart disease
CCQS: Cold Congealing and Qi Stagnation
QSBS: Qi Stagnation and Blood Stasis
DDA: Data dependent acquisition
HPLC-QTOF-MS: High-performance liquid chromatography coupled with quadrupole time-of-light mass spectrometry
HMDB: Human metabolome database
LIPIDMAPS: Lipid metabolites and pathways strategy
KEGG: Kyoto Encyclopedia of Genes and Genomes
VIP: Variable importance in the projection
PLS-DA: Partial least squares discriminate analysis
ATC: Anatomical Therapeutic Chemical
PPI: Protein–protein interaction
TCMSP: Traditional Chinese Medicine Systems Pharmacology Database and Analysis Platform
ETCM: Encyclopedia of traditional Chinese medicine
DPs: Differential proteins
DMs: Differential metabolites
AUC: Area under the receiver operating curve
